# Co-infection with Severe Fever with Thrombocytopenia Syndrome Virus and *Rickettsia japonica* after Tick Bite, Japan

**DOI:** 10.3201/eid2704.203610

**Published:** 2021-04

**Authors:** Tatsuya Fujikawa, Tomoki Yoshikawa, Takeshi Kurosu, Masayuki Shimojima, Masayuki Saijo, Kyoko Yokota

**Affiliations:** Mitoyo General Hospital, Kanonji, Japan (T. Fujikawa);; National Institute of Infectious Diseases, Tokyo, Japan (T. Yoshikawa, T. Kurosu, M. Shimojima, M. Saijo);; Kagawa Prefectural Central Hospital, Takamatsu, Japan (K. Yokota)

**Keywords:** severe fever with thrombocytopenia syndrome, severe fever with thrombocytopenia syndrome virus, Japanese spotted fever, tickborne infectious diseases, co-infection, rickettsia, vector-borne infections, bacteria, Japan, Rickettsia japonica, viruses

## Abstract

Severe fever with thrombocytopenia syndrome was diagnosed in a febrile woman in Japan after a tick bite. However, *Rickettsia japonica* DNA was retrospectively detected in the eschar specimen, suggesting co-infection from the bite. Establishment of the severe fever with thrombocytopenia syndrome virus infection might have overpowered the *R. japonica* infection.

Severe fever with thrombocytopenia syndrome (SFTS) is caused by SFTS virus (SFTSV), a novel phlebovirus in the family *Bunyaviridae* (*1*). It has been reported that SFTS is endemic to Japan (*2*). SFTS is classified as a viral hemorrhagic fever, and its case-fatality rate in Japan is ≈30% (*3*).

Japanese spotted fever (JSF) is an acute tickborne rickettsiosis caused by *Rickettsia japonica* and is endemic to Japan (*4*). Most cases of SFTS in Japan have been reported in southwestern Japan, and the JSF-endemic area overlaps the areas to which SFTS is endemic. Because the *Haemaphysalis longicornis* tick is a vector for both SFTSV and *R. japonica* (*4*,*5*), co-infection events might occur in patients with SFTS or *R. japonica* infection.

A woman 84 years of age was bitten on her lower right back by a tick while working in a field. She became febrile on day 1, experienced mild delirium on day 2, and visited the emergency department of Mitoyo General Hospital (Kanonji, Japan) on day 5, where she had low-grade fever but was alert and lucid. Physical examination revealed an eschar surrounded by exanthema on her lower right back (Figure). She had noticed the eschar on the day after the bite, and her family removed it. We observed no other skin exanthema on her body. Laboratory analysis revealed thrombocytopenia and leukocytopenia (Table). Serum chemistry analyses revealed elongation of the activated partial thromboplastin time and an increase in the D-dimer level, suggesting coagulopathy. Because increases in aspartate transaminase and blood urea nitrogen were noted, liver and renal functions might have been impaired transiently (Table).

**Figure:**
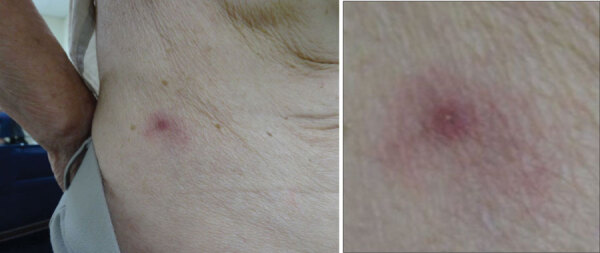
Eschar at site of tick bite surrounded by exanthema on lower right back of patient with severe fever with thrombocytopenia syndrome and positive *Rickettsia japonica* DNA in eschar, Japan.

**Table:** Laboratory findings in patient with severe fever with thrombocytopenia syndrome and positive *Rickettsia japonica* DNA in eschar at site of tick bite, Japan*

Parameter	Values	Reference range
Total blood cell counts		
Leukocytes	17.1 × 10^2^/µL	33–86 × 10^2^/μL
Erythrocytes	410 × 10^4^/µL	380–500 × 10^4^/µL
Hemoglobin	12.4 g/dL	11.5–15.0 g/dL
Hematocrit	35.2%	35.0%–45.0%
Platelets	7.4 × 10^4^/µL	15–35 × 10^4^/μL
Neutrophils	58%	35.0%–75.0%
Coagulation-associated tests		
PT	10.3 s	9.9–11.8 s
APTT	45.9 s	23.0–39.0 s
FDP	4.3 µg/mL	0.0–5.0 µg/mL
D-dimer	1.2 µg/mL	0.0–1.0 µg/mL
Serum chemistry		
CRP	<1.0 mg/dL	0.00–0.14 mg/dL
AST	42 U/L	13–30 U/L
ALT	18 U/L	7–23 U/L
ALP	170 U/L	106–322 U/L
γ-GT	14 U/L	9–32 U/L
Total bilirubin	0.5 mg/dL	0.4–1.5 mg/dL
LDH	200 U/L	124–222 U/L
CK	63 U/L	59–248 U/L
Total protein	7.1 g/dL	6.6–8.1 g/dL
Albumin	3.9 g/dL	4.1–5.1 g/dL
BUN	26 mg/dL	8–20 mg/dL
Creatinine	0.76 mg/dL	0.46–0.79 mg/dL
eGFR	54.5 mL/min	80–100 mL/min
Ccr	41.8 mL/min	NA
Uric acid	3.7 mg/dL	2.6–5.5 mg/dL
Sodium	127 mmol/L	138–145 mmol/L
Potassium	3.8 mmol/L	3.6–4.8 mmol/L
Chloride	92 mmol/L	101–108 mmol/L

*ALP, alkaline phosphatase; ALT, alanine transaminase; APTT, activated partial thromboplastin time; AST, aspartate transaminase; BUN, blood urea nitrogen; Ccr, calculated creatinine clearance; CK, creatine kinase; CRP, C-reactive protein; eGFR, estimated glomerular filtration rate; FDP, fibrin/fibrinogen degradation products; LDH, lactate dehydrogenase; PT, prothrombin time; SFTS, severe fever with thrombocytopenia syndrome; γ-GT, γ-glutamyl transpeptidase.

Because of the fever, thrombocytopenia, history of tick bite, and eschar with localized exanthema, we suspected JSF. The patient’s blood samples and the crust of the eschar were tested by PCR assays for *R. japonica*, *Orientia tsutsugamushi*, and SFTSV. The serum sample tested positive for SFTSV by a conventional 1-step reverse transcription PCR reported previously (*6*). *R. japonica* DNA was also detected in the eschar sample through the methods described previously (*7*), but it was not detected in serum samples. We empirically administered 100 mg of minocycline intravenously for 7 days, after which minocycline was administered orally every 12 hours for 3 days. Her symptoms resolved without complications by day 6, the second day of admission. After discharge from the hospital on day 12, outpatient follow-up was uneventful. 

We analyzed blood specimens to examine paired serum antibody titers against SFTSV in the acute phase and convalescent phase with indirect immunofluorescence assay (IFA) (*8*), which indicated a substantial increase in the antibody to SFTSV from <10 to 640. A relatively low level of viremia (154 copies/mL) was also confirmed in the acute phase (day 4) of the disease by quantitative PCR assays (6). We tested paired serum from the acute phase (day 4) and the convalescent phase (day 27) for IgG and IgM titers against *R. japonica* by IFA as described previously (*9*). IgG and IgM against *R. japonica* were not detected in either the acute-phase or convalescent-phase serum samples. This result suggests that a general *R. japonica* infection had not established itself and that infection was localized to the eschar, in which erythematous lesions were present, and *R. japonica* DNA was detected only in the eschar sample (Figure). Unfortunately, the nucleotide sequence of the *R. japonica* genome amplified from the eschar was not determined.

The clinical course and laboratory results of this patient, with the exception of the eschar, were consistent with SFTS but not JSF. It has been reported that a tick bite scar could not be found in 56% of SFTS patients (*6*), whereas skin eruptions appear in 100% of patients with JSF and tick bite eschar appear in 90% of patients with JSF (*10*). The patient showed no other skin eruptions besides the eschar at the site of the tick bite (Figure). It is highly possible that the eschar on this patient could have been caused by an inflammatory response induced by the local *R. japonica* infection. *R. japonica* did not induce systemic symptoms in this patient for 2 possible reasons. First, the incubation time for SFTS might be shorter than that of JSF. Second, the initiation of antimicrobial drugs in the early phase of disease might have ameliorated the clinical course of the diseases.

In conclusion, we describe a patient with a generalized SFTSV infection and a localized skin lesion caused by *R. japonica* at the site of a tick bite. This study suggests that SFTS patients with eschar at the site of a tick bite should be treated with appropriate antimicrobial drugs, such as doxycycline and minocycline.
